# Comparative metabolomics analysis of different resistant rice varieties in response to the brown planthopper *Nilaparvata lugens* Hemiptera: Delphacidae

**DOI:** 10.1007/s11306-019-1523-4

**Published:** 2019-04-11

**Authors:** Kui Kang, Lei Yue, Xin Xia, Kai Liu, Wenqing Zhang

**Affiliations:** 1 School of Life Sciences, Sun Yat-sen University, Guangzhou, 510275 Guangdong China; 2 Sun Yat-sen Memorial Hospital, Sun Yat-sen University, Guangzhou, 510275 Guangdong China

**Keywords:** Metabolomics, Rice (*Oryza sativa*), Spermidine, Quercetin, *Nilaparvata lugens*, Defence

## Abstract

**Introduction:**

The brown planthopper (BPH, *Nilaparvata lugens* Stål, Hemiptera: Delphacidae) is one of the most devastating insect pests of the crucially important cereal crop, rice (*Oryza sativa* L.). Currently, multiple BPH-resistant rice varieties have been cultivated and generalized to control BPH. However, the defence metabolic responses and their modes of action against BPH in different rice cultivars remain uncharacterized.

**Objective:**

We used a non-biased metabolomics approach to explore the differences in metabolite profiles in response to BPH infestation in the susceptible TN1 rice cultivar and two resistant cultivars (IR36 and IR56).

**Methods:**

The metabolomic detection based on gas chromatography–mass spectrometry (GC–MS) and liquid chromatography–mass spectrometry (LC–MS) was performed to investigate the content changes of identified metabolites in TN1, IR36 and IR56 rice varieties at various time points (0 h, 24 h, 48 h and 96 h) post BPH feeding. The differentially expressed metabolites were screened and the corresponding metabolic pathways were further enriched.

**Results:**

The results showed that compared to that in TN1, the content changes of most primary metabolites were more stable, but the concentration alterations of some defence-related metabolites were more acute and persistent in IR36 and IR56. Furthermore, the differentially expressed pathways analysis revealed that cyanoamino acids and lipids metabolism was persistently induced in IR36, but changes in thiamine, taurine and hypotaurine metabolism were more significant in IR56 during BPH infestation. Besides, the contents of quercetin and spermidine which were harmful to BPH fitness, were significantly elevated by BPH in TN1 and IR36, and the quercetin level was significantly decreased during BPH feeding in IR56.

**Conclusion:**

The results of the differences in metabolite profiles in response to BPH infestation in different rice cultivars were useful to clarify the metabolic mechanism of rice plants during BPH infestation and to provide new resources to control this insect pest.

**Electronic supplementary material:**

The online version of this article (10.1007/s11306-019-1523-4) contains supplementary material, which is available to authorized users.

## Introduction

Plants have developed multiple resistance strategies to defend themselves against attack from herbivorous insects, which could be generalized into constitutive and induced defences (Howe and Jander 2008; Schuman and Baldwin 2016). Constitutive defences, involving physical and chemical barriers, are active regardless of the presence of herbivores, whereas induced defences become activated only when plants are attacked by insects (Wu and Baldwin 2010; War et al. 2012). Compared to constitutive defences, plant-induced defences are usually thought to consume less resource and be more pest-specific, and hence have been the research focus for recent decades (Walling 2001; Chen 2008; Zhou et al. 2015). Upon insect attack, three primary events usually occur in order for host plants, from the recognition of pest feeding signals, the transmission of internal signals through the complex delivery network, to the production of multiple defensive compounds (Chen 2008; Fürstenberg-Hägg et al. 2013). As the end product of gene expression, plant metabolites, including primary metabolites and secondary metabolites, could participate in pest resistance by regulating the host’s basic biological activities or acting as insect toxins (Schwachtje and Baldwin 2008; Kessler 2015; Zhou et al. 2015).

A few examples of plant small molecules involved in the insect herbivory response include compounds such as most carbohydrates that provide essential energy needed for plant defence (Castrillón-Arbeláez et al. 2012; Zhou et al. 2015), aromatic amino acids (tryptophan, tyrosine and phenylalanine) that serve as precursors for the production of plant phytoalexin (Steinbrenner et al. 2011), and the richly diverse population of toxic plant secondary metabolites (Theis and Lerdau 2003). It has been estimated that the plant kingdom contains more than 200,000 metabolites and that each plant species or cultivated variety may contain its own chemotypic expression pattern (Dixon and Strack 2003; Windsor et al. 2005). Therefore, it is extremely challenging to determine the changes in plant metabolic state in response to insect herbivory using conventional chemical assays. Due to the rapid development of “omic” technologies and chemometric methodology, mass spectrometry (MS)—and nuclear magnetic resonance-based metabolomics approaches provide broader and nontargeted insight into metabolite profiling in host plants under biotic stress (Fiehn 2002; Hall 2006; Schauer and Fernie 2006). Metabolomics technologies have been widely applied in the study of plant–insect interactions (Widarto et al. 2006; Jansen et al. 2009; Marti et al. 2013; Lu et al. 2018). In combination with other omics sciences, such as transcriptomics or proteomics, metabolomics is becoming a key tool in the comprehensive understanding of the gene expression and metabolic regulation of plants to herbivory (Sumner et al. 2003; Schauer and Fernie 2006) and discovering the potential bioactive compounds that participate in plant–insect interactions (Jansen et al. 2009).

Rice (*Oryza sativa*) is a primary staple food for more than half of the world’s population and serves as the most human nutrition resource (Cheng et al. 2013). However, rice food security is seriously threatened by a variety of pests continuously. Among them, the brown planthopper (BPH), *Nilaparvata lugens* (Hemiptera: Delphacidae), a typical monophagous vascular feeder, has become the most destructive rice pest in many rice-growing regions (Cheng et al. 2013; Xue et al. 2014). For BPH control, host-plant resistance is generally considered to be an effective, economical and eco-friendly approach. Hundreds of BPH-resistant rice varieties have been cultivated and generalized by breeders since the 1960s, which have effectively suppressed the BPH populations and lower the rice yield losses in the last several decades (Qiu et al. 2011). It is believed that BPH resistance genes play a main role in the defence systems of resistant rice cultivars against BPH attacks, and to date, more than 30 BPH resistance loci have been reported in which eight genes have been successfully cloned. Many studies have revealed that introduced resistance genes could re-programming the expression patterns of genes, proteins and metabolites in resistant rice plants, and different BPH resistance genes endow rice cultivars with varied resistance mechanisms (Liu et al. 2010; Uawisetwathana et al. 2015). However, few studies have been performed to compare the difference in the defence mechanisms of resistant rice cultivars harbouring different BPH resistance genes on a metabolic level, which is important to uncover the resistance mechanisms of BPH resistance genes.

To strengthen our knowledge on the rice defence strategies against BPH attacks and to explore the distinction in metabolic response of different resistant rice cultivars to BPH attack, we used untargeted metabolomics to analyse the different metabolic patterns displayed in susceptible TN1 harbouring no BPH resistance gene, and two resistant rice varieties, IR36 carrying *bph2* and IR56 carrying *Bph1*, *bph2*, *Bph3* and *Bph32*, during BPH infestation. We had hypothesized that there was some difference in the response of primary metabolism and secondary metabolism to BPH infestation for three rice varieties harboring different BPH resistance genes. Subsequently, two secondary metabolites (quercetin and spermidine) were further selected to study their biological function in different rice varieties against BPH. The results promote the understanding of rice resistance mechanisms on the metabolic level, and provide novel insights for BPH population management.

## Materials and methods

### Plant and insect materials

The resistant IR36, IR56 and susceptible rice variety TN1 (used as a control in this study) were provided by the International Rice Research Institute (IRRI, Los Banos, the Philippines). All seeds were germinated on wet gauze placed in glass cups. Approximately 10 days later, the plantlets were transferred to larger pots (10 cm diameter) containing paddy field soil. Seedlings were then maintained in a greenhouse room at 26 ± 2 °C and 80 ± 10% humidity under a 16-h:8-h light:dark cycle. Rice leaf sheath at the five-leaf stage were used for experiments.

The BPH individuals used were collected from rice fields in Guangdong Province, China, and fed in a continuous laboratory culture on BPH-susceptible rice plants of Huang Hua Zhan (purchased from the Guangdong Academy of Agricultural Sciences, Guangzhou, China) for 8 years. The insects were maintained in the laboratory at 26 ± 2 °C with 80 ± 10% humidity and a light–dark cycle of L16 h:D8 h (Zhai et al. 2015).

### Herbivory treatments

One-day-old brachypterous females starved for at least 3 h prior to the start of the experiment were applied to each stem. Approximately 100 adult BPH insects (*N. lugens*) were introduced to per pot (an average of 10 adults per seedling) at 0 h, 48 h and 72 h after the beginning of the experiment, and each pot was covered by a plastic cage to prevent adults from escaping. All the rice samples were collected uniformly at 96 h after the first introduction of BPH (see Fig. S1). The rice plants which had been fed by BPH for 24 h was used for LC–MS analysis, and the samples fed by BPH for 24 h, 48 h and 96 h were used for GC–MS and qPCR analysis. The rice leaf sheath, including herbivore-exposed local (damaged) parts, was collected and used for analysis. There were six replications for each treatment and sampling time point. All 3 seedlings from each pot were collected as a replication, immediately placed into liquid nitrogen and then stored at − 80 °C.

### Extraction of rice metabolites

Metabolite extraction was performed using 100 mg of rice tissues according to the previously described method of Uawisetwathana et al. (2015), with the minor modifications. Briefly, 100 (± 1) mg of powdered plant tissues were extracted with 600 μL of a precooled methanol–water mixture (2:1) and sonicated for 15 min in an ice bath. Solutions were centrifuged at 20,000×*g* and 4 °C for 10 min before the supernatant was transferred to a new 1.5-mL Eppendorf tube. Then, the insoluble fraction was extracted repeatedly as the procedure above. The supernatant from the same sample was subsequently merged followed by 60 μL of an aqueous solution of ribitol (internal standard, 0.2 mg/mL, purchased from Sigma-Aldrich, A5502) was added. The resulting extract was concentrated to dryness under nitrogen. One part of the samples was used to perform the GC–MS analysis after derivatization, and the other samples were designated for LC–MS detection.

### GC–MS analysis

For the derivatization of rice metabolites, 70 μL of 20 mg/mL methoxy-amino-hydrochloride (Sigma-Aldrich) in pyridine was firstly added and the mixture were vortexed for 15 s. Subsequently, the reaction system was incubated for 1.5 h on a shaking table set at 37 °C and a rotating speed of 200 r/min. And then, 100 μL of *N*-methyl-*N*-(trimethylsilyl) trifluoroacetamide (MSTFA, Sigma-Aldrich) was added and shaken at 37 °C for 30 min. After centrifugation at 13,000×*g* for 3 min, 140 μL of the supernatant was collected and transferred into GC vials (2 mL, Agilent) for the next GC–MS detection.

The GC–MS detection for the derivatized samples was performed on an Agilent 7890A gas chromatograph equipped with an Agilent 5975C VL MSD detector (Agilent Technologies). 1 μL of the sample solution was firstly auto-injected into a DB-5Ms UI column (length = 30 m, I.D. = 25 mm, Agilent) by splitless sampling at 280 °C, and the analysis was conducted following the procedures as below: the oven temperature was initially held at 70 °C for 5 min, followed by rapidly increased to 300 °C at a rate of 5 °C/min and stable for 5 min. Helium was used as the gas carrier with the flow rate of 1 mL/min. Data were collected using the full scan mode across a mass range of 50–450 m/z, with the electron impact ionization (EI) energy of 70 eV.

GC–MS chromatograms were firstly acquired using the Agilent MSD Chemstation (version E.02.00.493), and retention time alignment was made using the online website of XCMS (https://xcmsonline.scripps.edu/) with the default parameters. And then the deconvolution and peak collection was further performed with the automated mass spectral deconvolution and identification system (AMDIS). To avoid false positives, the blank control was used to filter noisy signals and only the peaks with a signal-to-noise ratio (S/N) more than 30 were retained (Peng et al. 2016). Subsequently, the metabolites were identified by matching their mass spectra against the database of National Institute of Standards and Technology 8.0 (NIST, USA) according to the criteria described previously: match value ≥ 750, reverse match value ≥ 800, and a probability ≥ 60% (Zhu et al. 2016). The internal standard of ribitol was utilized to correct the retention time and calculate the concentrations of identified metabolites through relative peak area ratios. Finally, an assembled matrix including the metabolite abundance information for each sample was obtained, and was used in the next multivariate statistical analysis. The metabolites whose content changes were greater than twofold or less than 0.5-fold, and the p < 0.05 were thought as significantly differential substances.

### LC–MS analysis

The dried samples for the LC–MS detection were firstly resuspended in 50 μL chloroform. And then the LC–MS analysis was performed by an AB SCIEX TripleTOF 5600^+^ system with a Dionex Acclaim C18 2.6 μm, 2.0 × 150 mm column. The mobile phase consisted of 0.1% formic acid (FA) in water (phase A) and 0.1% FA in acetonitrile (phase B). The injection volume was 10 μL. A binary separation gradient was applied at a flow rate of 0.35 μL per min: 0–2 min, isocratic 95% A, 5% B; 2–10 min, linear gradient to 100% B; 11–12 min, isocratic 100% B; 12–15 min, linear gradient to 85% B. Process blanks (water only) and solvent blanks were used to remove artefactual peaks. Detection was performed by Q-TOF/MS in both electrospray negative (NI) and positive (PI) ion modes in independent runs with the following settings: capillary voltage at 4.5 kV(PI) and 3.75 kV (NI), cone voltage at 40 V, capillary exit at 100 V, desolvation temperature at 350 °C. The m/z range was 100–1000 Da with a scan time of 0.25 s. The MS was calibrated using sodium formate clusters occurring in 10 mM solution of NaOH in 50% v/v isopropanol/water with 0.2% formic acid. Data analysis was carried out by PeakView 1.6 and Markview (https://sciex.com/products/). Peak detection was performed using the ‘Peak Finding Options’ (minimum retention time = 2 min; minimum spectral peak width = 25 ppm; minimum RT peak width = 6 scans; noise threshold = 100). For alignments, ‘Alignment and Filtering’ was performed with list parameters: retention time tolerance at 0.5 min, mass tolerance at 10.0 ppm, removes peaks in two samples, maximum number of peaks at 8000. Peaks were identified based on the online database METLIN (https://metlin.scripps.edu/). Only the metabolites whose contents significantly increased or decreased were greater than twofold or less than 0.5-fold, and the p < 0.05 were selected to as the differential substances.

### RNA extraction and qPCR analysis

RNA was prepared from each repeat using TRIzol reagent (Invitrogen) following the manufacturer’s protocol and treated with DNase I (Takara Bio, Kyoto, Japan). Then, the cDNA was converted into first-strand cDNA using the PrimeScript™ 1st Strand cDNA Synthesis Kit (Takara Bio). Quantitative RT-PCR was performed using the Light Cycler 480 system (Roche Diagnostics, Indianapolis, IN, USA) and SYBR Premix Ex Taq (Roche Diagnostics) following the manufacturer’s protocol. The primers used for real-time PCR are listed in Table S1. The quantitative variation for each gene was calculated using a relative quantitative method $\left( { 2^{{ - \Delta \Delta {\text{C}}_{\text{T}} }} } \right)$ (Livak and Schmittgen 2001).

### Biological assay

The standard substances of quercetin (CAS: 117-39-5) and spermidine (CAS:124-20-9) were purchased from Sigma. Quercetin was diluted into 2, 10 and 50 ppm (parts per million) in artificial diet (Fu et al. 2001); in addition, spermidine was diluted into 1, 5 and 25 ppm. The rearing procedure is outlined in previous reports (Fu et al. 2001), and the length of glass cylinders was changed to 3 cm. Ten forth-instar individuals were placed in each glass cylinder as replicates, and we performed the experiments in triplicate for each gene. We counted the mortality every day. When the *N. lugens* individuals had developed into 2nd day adults, three brachypterous female adults from one chamber were randomly selected for body weight measurement, and the developmental duration was counted. Three biological replicates were performed.

### Statistical analysis

The multivariate statistical analysis for GC–MS samples were conducted on the MetaboAnalyst website (www.metaboanalyst.ca/). The raw data was firstly normalized with the concentration of internal standard substance of ribitol, and then all the data was transformed by log transformation, scaled by auto scaling (mean-centered and divided by the standard deviation of each variable). The principal component analysis (PCA) was firstly performed to investigate the relationships among the test samples. Next, the variable importance in projection (VIP) value was calculated to assess the contribution of each differential metabolite in representing the difference of rice resistance, by comparing the metabolite profiling of resistant rice (IR36 or IR56) and susceptible TN1 samples at the same time point post BPH-feeding. The metabolites of VIP value greater than 1 from each time point were further filtered, and their intersection was finally acquired using Venn diagrams. For statistical analysis of the LC–MS results, the relative expression was calculated by the intensity of two groups. And the differences between two groups were analysed using t-tests.

The differentially expressed metabolic pathways in three rice varieties at different time points during BPH infestation were acquired by mapping the differential metabolites to their respective biochemical pathways using MetPA online tools (http://metpa.metabolomics.ca/). Through a hypergeometric test, the metabolic pathways whose log(− p) value greater than 1.301 (p < 0.05) were lastly retained.

The effect of BPH feeding on the bioactive substances (quercetin and spermidine) levels and related gene expression changes in rice plants were analysed with ANOVA (*α* = 0.05, Duncan) using SPSS 18.0. The same statistical analysis method was also used to explore the influence on BPH fitness (mortality, body weight and developmental duration) of feeding on artificial diets containing quercetin or spermidine.

## Results

### Primary metabolic response of different rice cultivars to BPH feeding determined by GC–MS

Using AMDIS software, 1200 peaks were detected from the GC–MS spectra of all the rice samples. By comparing with the blank samples and removing the noise peaks (S/N < 30), approximately 120 substances were finally identified through a NIST 08 database search. Subsequently, a total of 60 metabolites that were identified in all the GC–MS samples were acquired (Table S2). Most of them were primary metabolites, including 14 amino acids, 12 sugars or sugar alcohols, 12 organic acids, 6 fatty acids, 4 nucleotides, 4 sterols, 4 vitamins and 4 other chemical compounds.

**Fig. 1:**
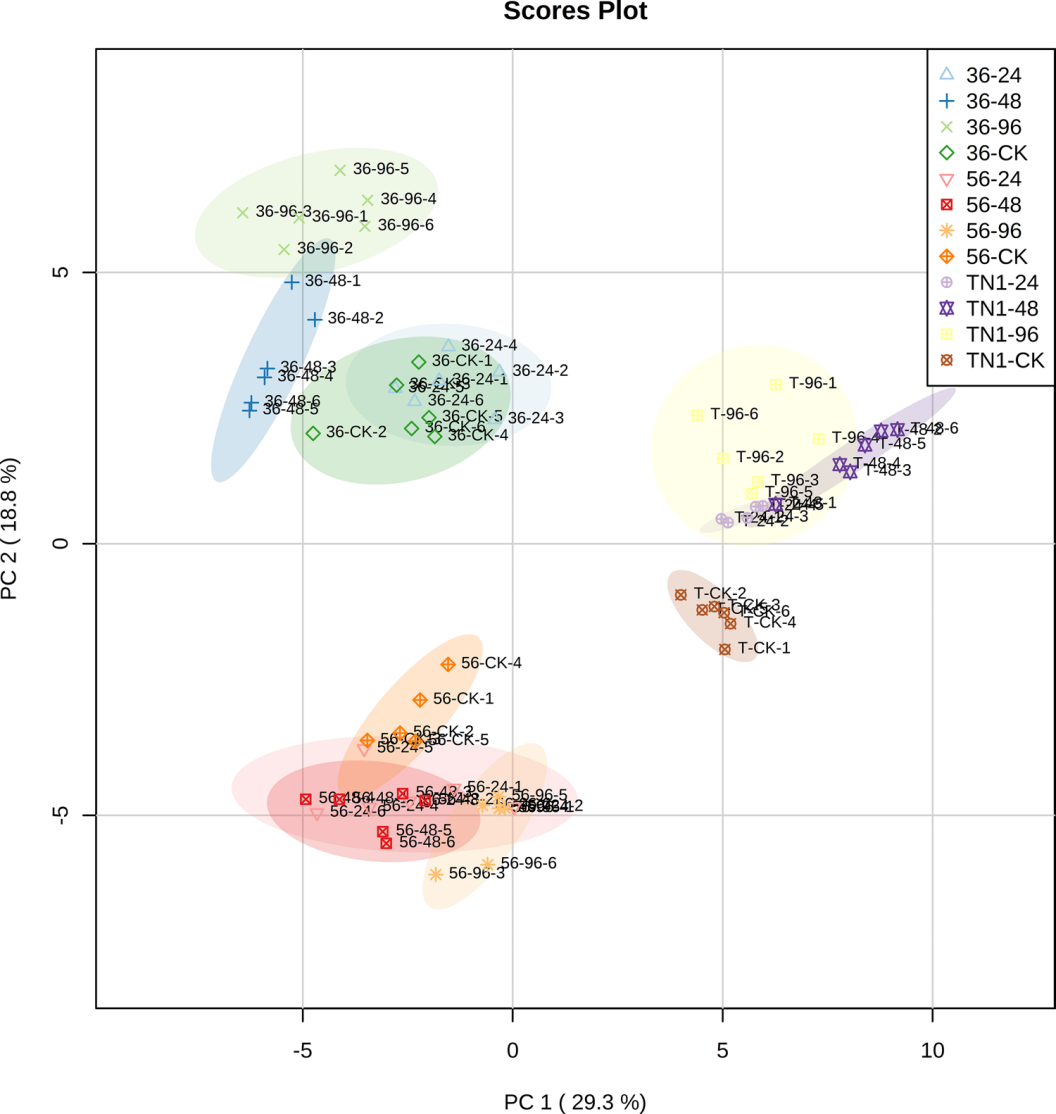
PCA score plot of leaf sheath metabolic profiles measured by GC–MS. The rice samples from three rice varieties (TN1, IR36 and IR56) were infested by BPH for 24 h, 48 h and 96 h. *36*–*24* represents IR36 rice plants infested by BPH for 24 h, *36*–*48* represents IR36 rice plants infested by BPH for 48 h, *36*–*96* represents IR36 rice plants infested by BPH for 96 h, and *36*-*CK* represents IR36 rice plants without BPH infestation. 5*6*–*24* represents IR56 rice plants infested by BPH for 24 h, *56*–*48* represents IR56 rice plants infested by BPH for 48 h, *56*–*96* represents IR56 rice plants infested by BPH for 96 h, *56*-*CK* represents IR56 rice plants without BPH infestation. *TN1*-*24* represents TN1 rice plants infested by BPH for 24 h, *TN1*-*48* represents TN1 rice plants infested by BPH for 48 h, *TN1*-*96* represents TN1 rice plants infested by BPH for 96 h, *TN1*-*CK* represents TN1 rice plants without BPH infestation

Principal component analysis (PCA) was performed to evaluate the effect of BPH infestation and rice types on the metabolic profiles in rice leaf sheaths (Fig. 1). The first two components (PC1 and PC2) accounted for 48.1% of the total variation in the PCA score plot of rice samples (PC1, 29.3%; PC2, 18.8%). The results showed that the metabolic profiles of different rice samples could be clearly separated into three main groups in terms of rice cultivars, and subgroups were also further defined based on BPH treatments within each rice variety (Fig. 1). The results suggested that both the factors of rice types and BPH infestation times could have significant impacts on rice metabolic profiles, whereas the effect of rice cultivars was more obvious.

The variable importance in the projection (VIP) scores were further calculated to evaluate the contributions of identified metabolites on group separations observed in the PCA plot, and the metabolites whose VIP values were greater than 1 were filtered. The results showed that some sugars (such as fructose, glucopyranose and raffinose), fatty acids (such as octadecadienoic acid, octacosanoic acid and triacontanoic acid) and amino acids (such as proline, norleucine and phenylalanine) had the most concentration variations during BPH infestation in the TN1 rice variety (Table S3). However, for resistant rice varieties IR36 and IR56, in addition to the above primary substances, some secondary metabolites or their precursor substances also contributed the most to the differences in rice metabolic profiles at different time points post-BPH infestation (Table S3). In this study, the concentrations of some flavonoids (for example, loganin), polyamines (for example, putrescine) and non-protein amino acids (for example, 3-cyano-alanine) were mostly influenced in the IR36 rice cultivar during BPH infestation, whereas some metabolites located on the phenylpropanoid secondary metabolic pathway, including shikimic acid, 4-hydroxy-cinnamic acid and cinnamate, contributed the most to the changes in rice metabolic profiles post-BPH feeding.

The concentration changes of metabolites contained in three rice cultivars during BPH infestation were further compared by categories (Table S2). The results showed that BPH feeding elevated most non-essential amino acid levels in susceptible TN1 cultivars but lowered the concentrations in most essential amino acids, such as lysine, isoleucine, leucine and valine, in resistant rice varieties (Fig. S3). The content of phenylalanine, which is one of most important precursors of secondary metabolism, was significantly elevated by BPH infestation in TN1 and IR36 rice cultivars but had no changes in IR56 cultivar. For carbohydrate metabolism, most sugar levels were downregulated for a short amount of time by BPH feeding for all rice cultivars, but with an extension of BPH infestation, the contents of many sugars (e.g., glucose and maltose) recovered in resistant rice varieties IR36 and IR56 but not in the susceptible TN1 cultivar. In addition, compared to that in resistant rice cultivars, the concentrations of some intermediate metabolites in TCA recycling (such as citric acid, malic acid and α-ketoglutaric acid) were significantly decreased in susceptible TN1 after long-time (96 h) feeding (Fig. S3), suggesting that the energy supply, which was crucial for BPH defence, was suppressed in this susceptible rice cultivar. Moreover, the levels of shikimic acid and cinnamic acid, which are two precursor substances of secondary metabolites in plants, were also significantly lowered in the TN1 cultivar after 96 h feeding by BPH.

The Venn diagram of changing metabolic pathways induced by BPH infestation in three different rice varieties showed that there were five metabolic pathways shared in the three rice varieties responding to BPH infestation, involving the metabolism of some basic compounds, such as alanine, aspartate, galactose and ubiquinone, suggesting that the primary metabolism was profoundly impacted by BPH feeding in all three rice cultivars (Table 1). However, by comparing the changes in metabolic pathways responding to BPH infestation in different rice varieties, it showed that in addition to TN1 rice cultivars, the metabolism of some non-protein amino acids (such as some cyanoamino acid, taurine and hypotaurine), vitamins and lipids were consistently affected in resistant IR36 and IR56 rice cultivars. Furthermore, lipid metabolism was exclusively induced by BPH feeding in the IR36 rice cultivar, whereas thiamine metabolism was more significant in the IR56 rice cultivar in response to BPH attack, displaying different metabolic responses to BPH feeding in different rice varieties.

**Table 1:** List of changed metabolic pathways responding to BPH infestation in three rice varieties

Intersections	Changed metabolic pathways induced by BPH feeding^a^
All	Galactose metabolism
	Alanine, aspartate and glutamate metabolism
	Starch and sucrose metabolism
	Ascorbate and aldarate metabolism
	Ubiquinone and other terpenoid-quinone biosynthesis
TN1 and IR36	Valine, leucine and isoleucine biosynthesis
	Phenylalanine, tyrosine and tryptophan biosynthesis
	Biotin metabolism
	d-Glutamine and d-glutamate metabolism
TN1 and IR56	–^b^
IR36 and IR56	–
TN1 exclusively	Pentose and glucuronate interconversions
	Glycine, serine and threonine metabolism
	Nitrogen metabolism
	Phenylalanine metabolism
IR36 exclusively	Arginine and proline metabolism
	Amino sugar and nucleotide sugar metabolism
	Cyanoamino acid metabolism
	Glycerolipid metabolism
IR56 exclusively	Taurine and hypotaurine metabolism
	Thiamine metabolism

^a^The continuously induced metabolic pathways at 24 h, 48 h and 96 h across BPH feeding in three rice varieties were listed as above

^b^Indicates there is no common pathways between corresponding rice varieties

### Secondary metabolic responses of different rice cultivars to BPH feeding for 24 h determined by LC–MS

From the results acquired from GC–MS, we found that metabolic defence was adequately induced by 24 h feeding by BPH in all rice varieties, and hence, LC–MS analysis was further performed to explore the secondary metabolic responses in different rice varieties to BPH attack at 24 h. By pre-processing the data, the information on the MS/MS spectra and corresponding retention times of 1071 individual features were acquired. Subsequently, we searched the above features in the Formula Finder 3.0 database, and 153 potential substances were obtained. Then, with the help of the METLIN database, 105 metabolites were identified.

By counting the metabolites whose contents were significantly changed at 24 h BPH infestation in different rice varieties, it was found that the number of metabolites whose concentrations were significantly increased in IR56 was 88, which was much more than that in TN1 and IR36 rice cultivars (Table 2). In contrast, only 54 metabolites were significantly decreased in IR56 infested by BPH, while the number in susceptible TN1 and resistant IR36 was 70 and 79, respectively (Table 2).

**Table 2:** Number of changed metabolites induced by 24 h BPH feeding in three rice varieties identified using LC–MS

	TN1	IR36	IR56
Up twofold	46	46	88
Down twofold	70	79	54
Identified number	24	16	23

We subsequently determined the content changes of the identified compounds in three rice varieties in detail. The results showed that only one substance, 2-naphthylamine, was significantly induced in all three rice varieties, and 7 metabolites were affected in two varieties, including bendiocarb, quercetin and telmisartan in TN1 and IR56; olsalazine and spermidine in TN1 and IR36; and acebutolol and sertindole in IR36 and IR56, post-24 h BPH feeding (Table S4). In addition, there were 18, 11, and 17 metabolites that were changed in only TN1, IR36 and IR56, respectively (Table S4). The LC–MS results also showed some differences in the influenced metabolic pathways of three rice varieties responding to BPH infestation. In TN1, β-alanine metabolism was exclusively inhibited by BPH feeding, whereas glutathione metabolism was significantly induced in the IR36 rice cultivar. In addition, flavone and flavonol biosynthesis and thiamine metabolism were significantly changed in IR56 during BPH infestation.

### BPH feeding altered quercetin and spermidine levels in all three rice varieties

**Fig. 2:**
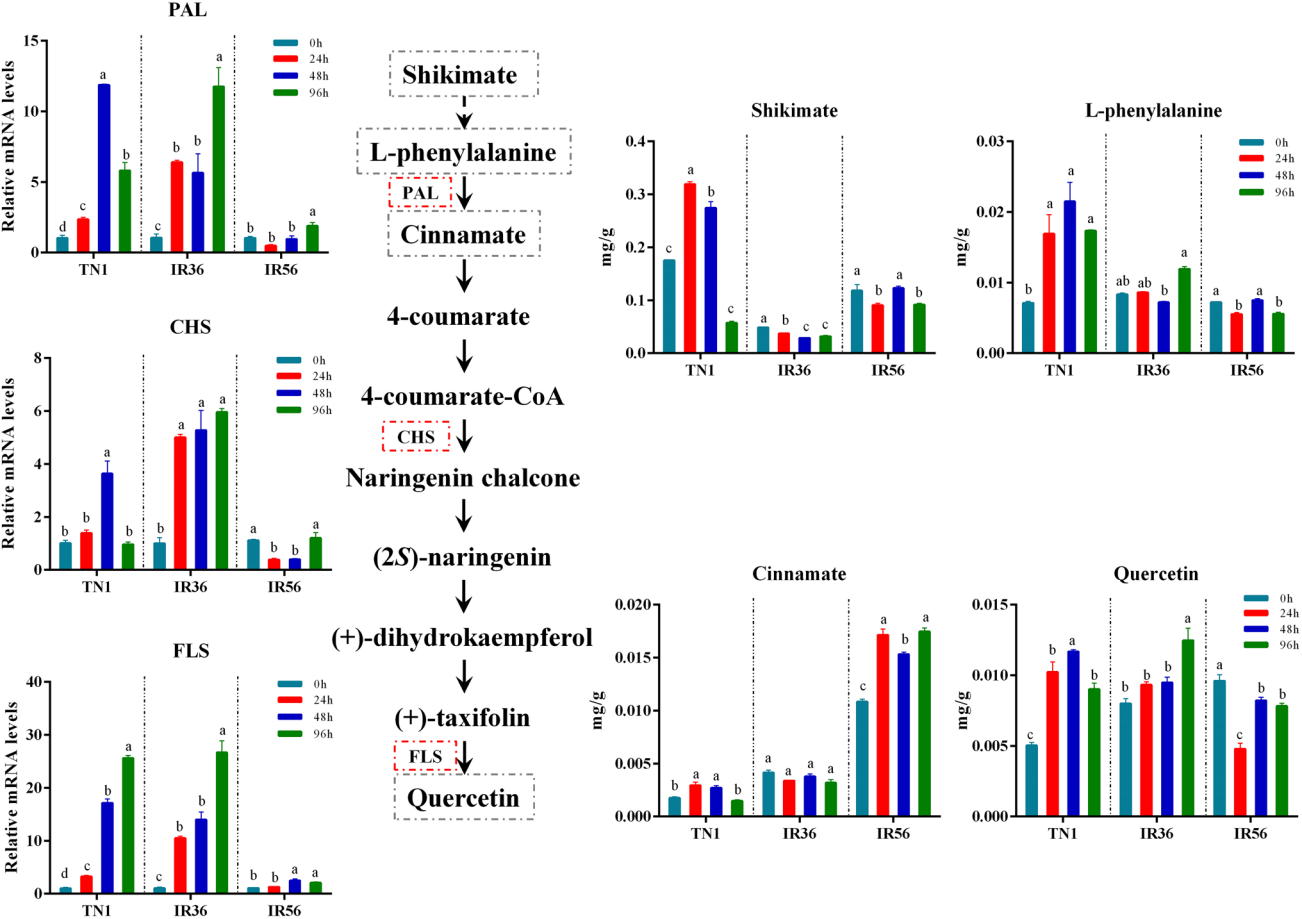
Abundance of metabolites and expression pattern of genes of the quercetin biosynthesis pathways during BPH infestation. The x-axis represents the rice cultivar in which different rice varieties were separated by dotted lines, while the y-axis represents the metabolite abundance or gene expression level. *PAL* is a gene encoding phenylalanine ammonia-lyase, *CHS* is a chalcone synthase gene and *FLS* is a flavonol synthase gene. Rice *Actin1* was used as a reference control. The expression of genes was quantified relative to the values obtained from 0 h samples from corresponding rice varieties. In all panels, only the values from the same rice varieties were compared (different letters indicate statistically significant difference, *p *< 0.05, by ANOVA). All the values are shown as the mean ± SEM

**Fig. 3:**
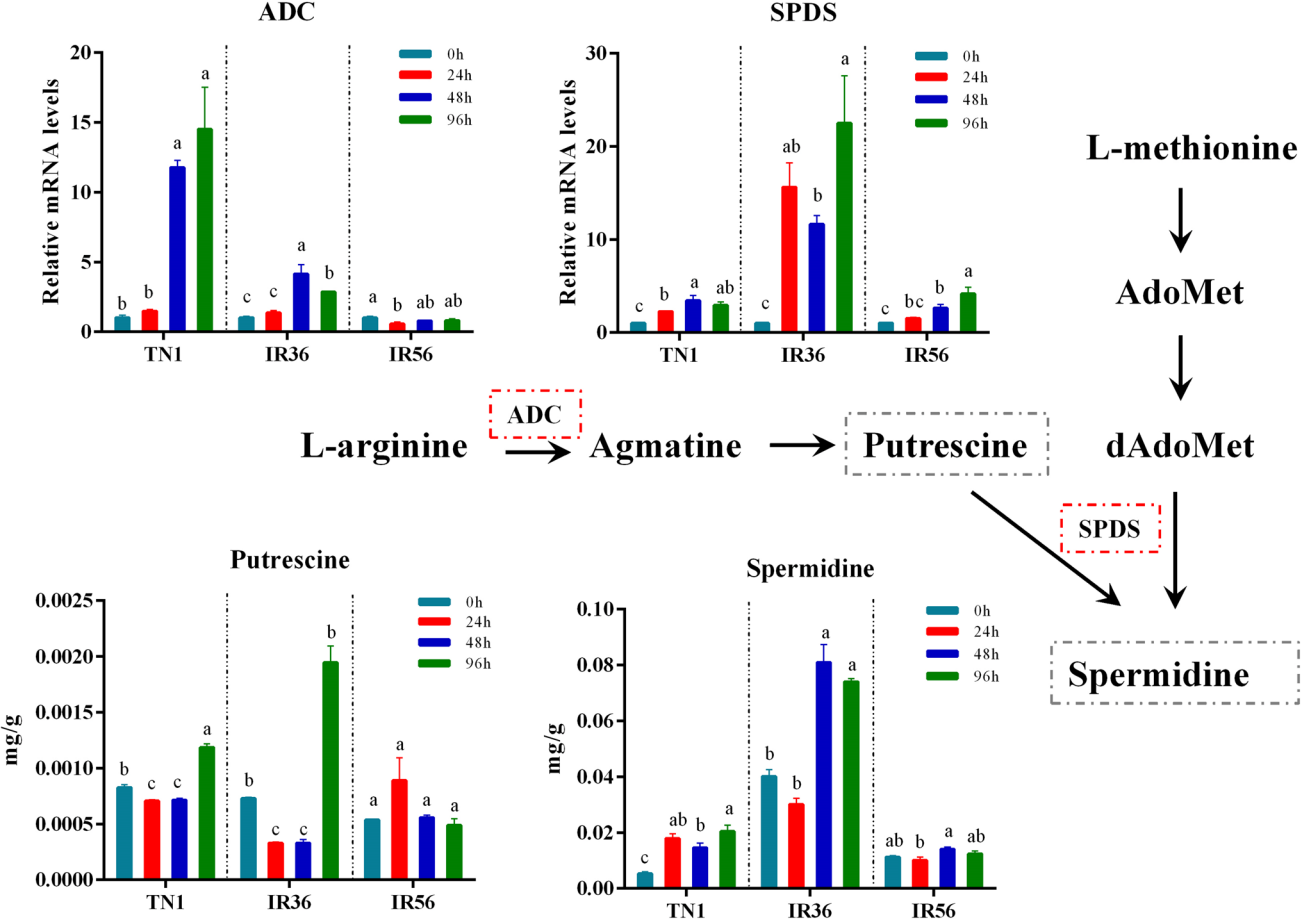
Abundance of metabolites and expression pattern of genes of spermidine biosynthesis pathways during BPH infestation. The x-axis represents the rice cultivar in which different rice varieties were separated by dotted lines, while the y-axis represents the metabolite abundance or gene expression level. *ADC* is an arginine decarboxylase gene, and *SPDS* is a spermidine synthase gene. Rice *Actin1* was used as a reference control. The expression of genes was quantified relative to the values obtained from 0 h samples from corresponding rice varieties. In all panels, only the values from the same rice varieties were compared (different letters indicate statistically significant difference, *p *< 0.05, by ANOVA). All the values are shown as the mean ± SEM

By analysing the quercetin and spermidine accumulations in different rice cultivars after BPH infestation, we found that BPH induced the accumulation of the two metabolites in the TN1 variety (*p *< 0.05, ANOVA, Fig. 2; Fig. 3), implicating the positive responses of rice plants to BPH feeding. In addition, the quercetin and spermidine levels were also significantly elevated at 96 h BPH infestation in the IR36 cultivar (*p *< 0.05, ANOVA, Fig. 2; Fig. 3). However, in the IR56 cultivar, BPH feeding significantly reduced the quercetin content at all feeding points (*p *< 0.05, ANOVA, Fig. 2), and the spermidine accumulation showed no significant changes at any measurement point (*p *< 0.05, ANOVA, Fig. 3), which might be the result of host manipulation by BPH herbivory.

To determine the expression changes of genes that function in quercetin and spermidine synthesis in different rice cultivars, qPCR experiments were performed. The results showed that at most of the BPH feeding stages, the expression levels of quercetin synthesis-related genes, including the chalcone synthase gene (*CHS*), phenylalanine ammonia-lyase gene (*PAL*) and flavonol synthase gene (*FLS*), were significantly elevated by BPH feeding in TN1 and IR36 rice varieties (*p *< 0.05, ANOVA, Fig. 2), but 24 h and 48 h feeding significantly lowered the expression of *CHS* in IR56 rice plants (*p *< 0.05, ANOVA, Fig. 2). With a similar alteration pattern, the expression levels in two spermidine synthesis-related genes, including the arginine decarboxylase gene (*ADC*) and the spermidine synthase gene (*SPDS*), were also significantly upregulated during BPH infestation in the TN1 and IR36 rice cultivars (*p *< 0.05, ANOVA, Fig. 3), while *SPDS* expression was significantly decreased at 24 h post-BPH feeding in the IR56 rice variety (*p *< 0.05, ANOVA, Fig. 3).

### Ingestion of quercetin and spermidine lowered the BPH fitness

**Fig. 4:**
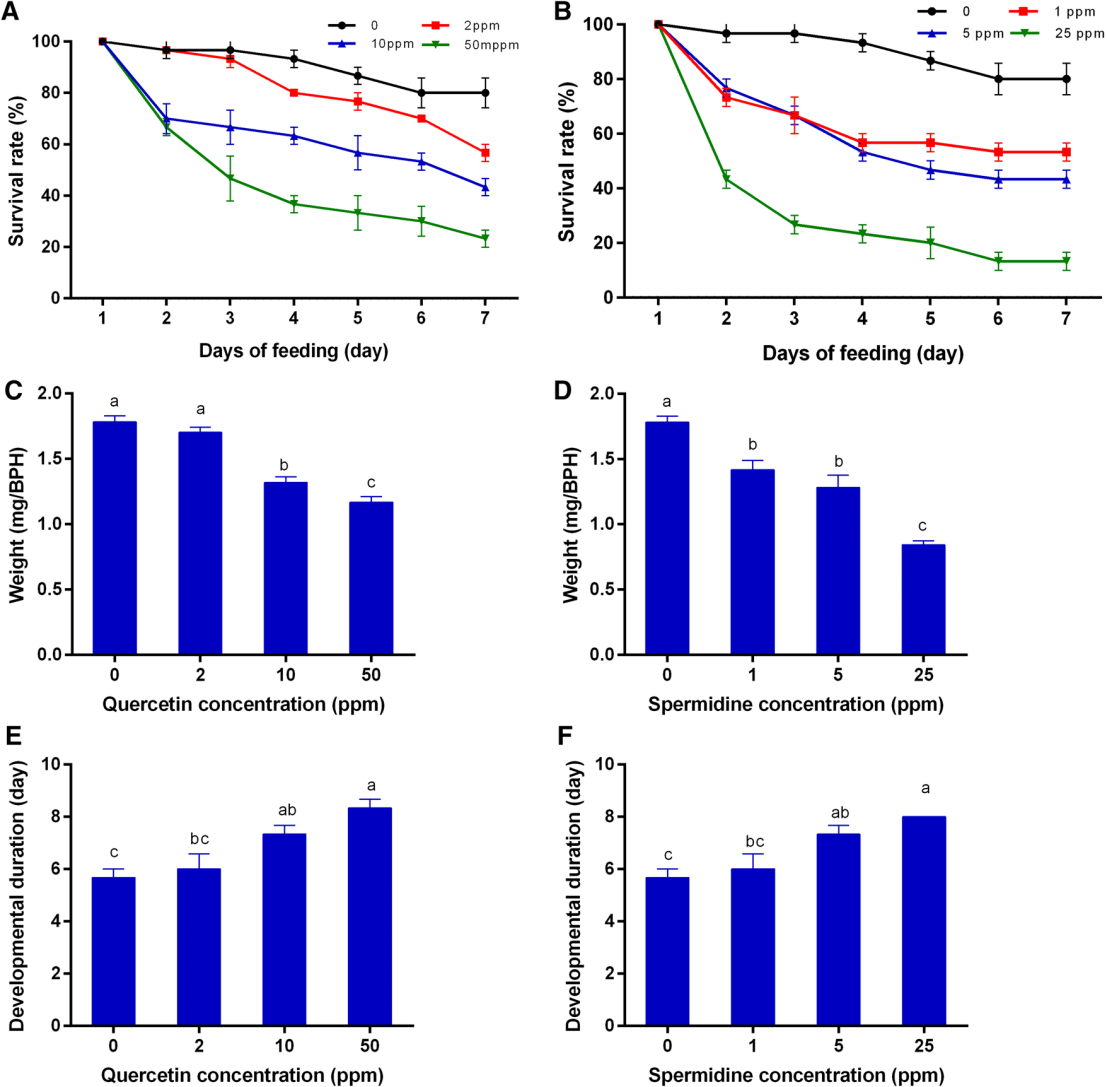
Influence of feeding artificial diets containing quercetin or spermidine at different concentrations on BPH fitness. **a** Impact of ingesting artificial diets containing quercetin on BPH survival; **b** impact of ingesting artificial diets containing spermidine on BPH survival; **c** impact of ingesting artificial diets containing quercetin on BPH body weight; **d** impact of ingesting artificial diets containing spermidine on BPH body weight; **e** impact of ingesting artificial diets containing quercetin on BPH developmental duration; **f** impact of ingesting artificial diets containing spermidine on BPH developmental duration. Values with different letters are significantly different by the Duncan’s multiple range test (*p* < 0.05). All the values are shown as the mean ± SEM

Feeding on the artificial diets containing quercetin or spermidine significantly increased the BPH mortality during the development from 4th nymph to 2nd day adult with the dose effect (Fig. 4a, b). The body weights of the 2nd day adults in the treatments and controls showed significant differences and was reduced to 65.2% and 47.2% when fed quercetin and spermidine, respectively (Fig. 4c, d). In addition, developmental duration from the 4th nymph to the 2nd day adult was prolonged from 5.8 to 47.1% when three concentrations of quercetin were added to the artificial diets (Fig. 4e). Meanwhile, feeding on three concentrations of spermidine added to artificial diets prolonged the developmental duration from 5.8 to 41.2% (Fig. 4f). In conclusion, quercetin and spermidine decreased the fitness of BPH.

## Discussion

BPH feeding can broadly interfere with host physical metabolism and rice plants could also change their metabolic patterns to combat BPH attack and maintain normal biological activities (Wang et al. 2005; Liu et al. 2010; Uawisetwathana et al. 2015; Alamgir et al. 2016). Therefore, the rice metabolic profiles during BPH infestation represent the outcomes of BPH-rice interactions. The physiological changes caused by BPH feeding in different rice cultivars are related to BPH resistance level or BPH resistance genes (Liu et al. 2010; Chen et al. 2012; Cheng et al. 2013). The resistant rice varieties of IR36 and IR56 used in our research were bred by IRRI and had been widely applied in the southeast Asia to suppress BPH population. These two rice varieties harbored different BPH resistance genes, in which IR36 cultivar carried *bph2* and IR56 cultivar carried multiple resistance genes, including *Bph1*, *bph2*, *Bph3* and *Bph32* (Fujita et al. 2013; Hu et al. 2016; Yue et al. 2018). Many studies had showed that infestation of IR36 and IR56 rice plants had a significant negative effect on BPH fitness, and the BPH resistance level owned by IR56 rice were much higher than that of IR36 variety (Velusamy and Heinrichs 1986; Wu et al. 2009). In this study, three rice varieties with different BPH resistance levels displayed different metabolic patterns at 24 h, 48 h and 96 h post-BPH infestation, which might partly be attributed to the different BPH resistance genes they harbour.

The primary metabolism was broadly inhibited in all three rice varieties throughout BPH infestation, including sugar, amino acid and vitamin metabolism (Table S2), suggesting that BPH feeding could have profound inference on the metabolic profiles of these three rice cultivars, which had been also reported in other rice varieties (Liu et al. 2010; Uawisetwathana et al. 2015). The primary metabolites are not only the main nutrition and energy source of host plants and insect herbivores (Cockfield 1988; Bruyn et al. 2002), but also provide the essential fuel and raw materials for the secondary metabolisms in plants combating pest infestation (Hanik et al. 2010; Steinbrenner et al. 2011; Castrillón-Arbeláez et al. 2012). In current study, although short-time (24 h) feeding by BPH caused notable reductions in the concentrations of most sugars (such as, glucose, fructose and maltose) in all three rice cultivars, most sugars levels recovered in IR36- and IR56-resistant rice cultivars at 96 h post BPH feeding, indicating the physiological adjustment ability in sugar metabolism was stronger in resistant rice cultivars IR36 and IR56 than in the susceptible TN1 variety. Similarly, the concentrations of other primary metabolites, such as most amino acids, organic acids and fatty acids, were also more stable in resistant rice varieties in this study (Fig. S3; Table S2). Apparently, the stability of most basic metabolite levels was favourable for maintaining normal growth for IR36 and IR56 rice cultivars under feeding stress from BPH herbivores, displaying a higher tolerance for BPH, which is common in many resistant rice varieties (Qiu et al. 2011).

The key roles of plant secondary metabolites (PSM) defence against herbivores have been widely recognized (Kessler 2015; Züst and Agrawal 2016). PSM can significantly lower herbivore fitness through direct defence functions (e.g., disturbing the herbivore’s food-intake, reducing food-digestibility or producing direct toxic compounds) or indirect defence functions (e.g., attraction of natural enemies of pests) (Kessler 2015). In this study, compared to the susceptible TN1 rice cultivar, some secondary metabolites, including flavonoids, polyamines and non-protein amino acids, or their precursor substances played more important roles in discriminating the rice metabolic profiling induced by BPH feeding across various time points in resistant rice cultivars (Table S3). Further analysis also showed that the concentration alterations of some secondary metabolites (such as fenazaquin, telmisartan and milbemectin A4) (Table S4) and precursor substances (such as putrescine, shikimic acid, cinnamic acid) were more acute and persistent in the resistant IR36 and IR56 cultivars (Fig. S3), suggesting that the secondary metabolism activation increased with BPH feeding in resistant IR36 and IR56 rice cultivars, which might contribute to higher resistance levels against BPH herbivores. Similar metabolic responses to BPH attack were also reported in the resistant rice variety B5 by Liu et al. (2010).

In addition, there were also some differences in the metabolic responses to BPH attack between the IR36 and IR56 cultivars, which was probably due to the difference of BPH resistance genes they harboured. For example, the metabolisms in cyanoamino acids and lipids were exclusively induced in the IR36 rice cultivar during BPH infestation, while taurine, hypotaurine and thiamine were significantly elevated by BPH feeding in the IR56 rice cultivar (Table 1). The upregulated expression of non-protein amino acids observed in the IR36 rice cultivar has been widely reported to be unfavourable to the fitness of insect herbivores (Huang et al. 2011), and changes in lipid metabolism might be helpful in adding the energy expended in BPH defence (Lim et al. 2017). However, in IR56 rice plants, the accumulation levels of taurine, hypotaurine and thiamine were notably increased during BPH infestation, which might function in relieving the ROS-induced oxidative pressure caused by BPH feeding (Ahn et al. 2005; Liu et al. 2010; Uawisetwathana et al. 2015).

By analysing the concentration changes in detected metabolites in different rice cultivars caused by BPH attack, the substance levels of phenylpropanoid metabolism and polyamine metabolism, which have been widely revealed to play crucial roles in plant defence against herbivores (Turner et al. 2012; Jiménez-Bremont et al. 2014), displayed different patterns of change in three rice varieties. Therefore, two downstream products of quercetin and spermidine, whose contents greatly changed at 24 h post-BPH feeding in three rice cultivars revealed by LC–MS results (Table S4), were further selected to study their biological function in different rice cultivars against BPH.

Spermidine- or quercetin-supplemented food was shown to lower the fitness of BPH (Fig. 4), indicating that spermidine and quercetin were resistance-related metabolites in rice defence against BPH. Previous studies have shown that quercetin could function as an activator to improve plant resistance against phytopathogens (Taguri et al. 2006; Maddox et al. 2010) and was modulated by the salicylic acid (SA)-dependent pathway from an early stage upstream of *NDR1* and *EDS1* (Yang et al. 2016). Spermidine confers resistance to rice blast accompanied by the upregulation of marker genes for the salicylic acid-mediated signalling pathway *PR1b* and *PBZ1* and of phytoalexin biosynthesis genes *CPS4* and *NOMT* (Moselhy et al. 2016). In this study, quercetin accumulation was significantly increased in susceptible TN1 rice after BPH treatment and decreased in IR56, while there were no significant changes in IR36 post-96 h infestation (Fig. 2). Another secondary metabolite, spermidine, was also significantly increased in susceptible TN1 and resistant IR36 after BPH treatment, while there were no significant changes in IR56 (Fig. 3). These differences might be due to the different resistance genes harboured by those two rice cultivars. The IR36 variety carried the resistance gene of *bph2* (thereafter renamed *BPH26*), which was derived from ASD7 (Jena and Kim, 2010). Some studies showed that *bph2* could induce BPH sucking inhibition in the phloem sieve element and could be considered to be receptors related to signal perception and transduction (Tamura et al. 2014). IR56 possesses multiple resistance genes (Yue et al. 2018), containing *Bph3,* derived from Rathu Heenati, which might induce the pattern-triggered immunity response to BPH infestation by perceiving herbivore-associated molecular patterns (HAMPs) or damage-associated molecular patterns (DAMPs), or mediating the downstream signaling events (Liu et al. 2015), whereas *BPH26* promotes rice resistance to BPH by inducing the SA signalling pathway (Cheng et al. 2013; Tamura et al. 2014).

Through long-term evolution and natural selection, insects have developed adaptive mechanisms to evade plant defence mechanisms (Zhu-Salzman et al. 2005). Host manipulation is one of the most important counter-defence strategies employed by insects (Karban and Agrawal 2002; Kojima et al. 2010). By re-programming host metabolic profiles, herbivores could acquire more nutrients (Sandstrom et al. 2000; Koyama et al. 2004; Oliveira et al. 2016) or downregulate the host defence (Consales et al. 2012, p. 20. Hattori et al. 2012; Elzinga and Jander 2013). For instance, *Nephotettix cincticeps* salivary secretion contains a novel calcium binding protein that might prevent sieve-element occlusion to promote continuous feeding (Hattori et al. 2012). As an inhibitor of phloem sap, the concentration of tricin was significantly higher in fresh leaves of resistant rice Rathu Heenati (RHT) than that of the susceptible rice variety TN1 (Zhang et al. 2015). However, after BPH feeding, the tricin concentration was decreased in RHT and increased in TN1 (Zhang et al. 2015), suggesting that BPH might regulate the metabolic accumulation in rice by salivary proteins (Ji et al. 2017). In our study, spermidine and quercetin had similar expression patterns as tricin, and BPH had a stronger inhibitory effect on the expression of these two substances in IR56. Although previous research suggested that the resistance of IR56 to BPH was stronger than that of IR36 (Zheng et al. 2016), the decrease in spermidine and quercetin levels was more obvious in IR56 rice variety (Fig. 2; Fig. 3), which were seemingly more favourable to BPH fitness. For this paradox, we will say that BPH resistance in rice varieties is the comprehensive effect of various kinds of secondary metabolites. Further work is needed to explore the resistance mechanisms against BPH in IR36 and IR56 rice varieties.

## Conclusions

Using a non-biased metabolomics approach, we found that compared to that in susceptible TN1 rice variety, the level changes of most primary metabolites were stable and concentration alterations of defence-related metabolites were more acute and persistent in resistant IR36 and IR56 varieties during BPH infestation. And metabolic pathways were differentially expressed in IR36 and IR56 caused by BPH. Besides, the content changes of quercetin and spermidine which were harmful to BPH fitness, exhibited different pattern in three rice cultivars responding to BPH attacks. The results were useful to clarify the metabolic mechanism of rice plants during BPH infestation and to provide new resources to control this insect pest.

## Electronic supplementary material

Below is the link to the electronic supplementary material.

Supplementary material 1 (XLSX 36 kb)

Supplementary material 2 (DOCX 364 kb)
