# Supplementary material for: Comparative metabolomics analysis of different resistant rice varieties in response to the brown planthopper Nilaparvata lugens Hemiptera: Delphacidae
Source: Metabolomics. 2019 Apr 11;15(4):62. doi: 10.1007/s11306-019-1523-4 (PMC6459800; doi:10.1007/s11306-019-1523-4)
Supplement: Supplementary file 2 — Supplementary material 2 (DOCX 364 kb) [file 11306_2019_1523_MOESM2_ESM.docx]

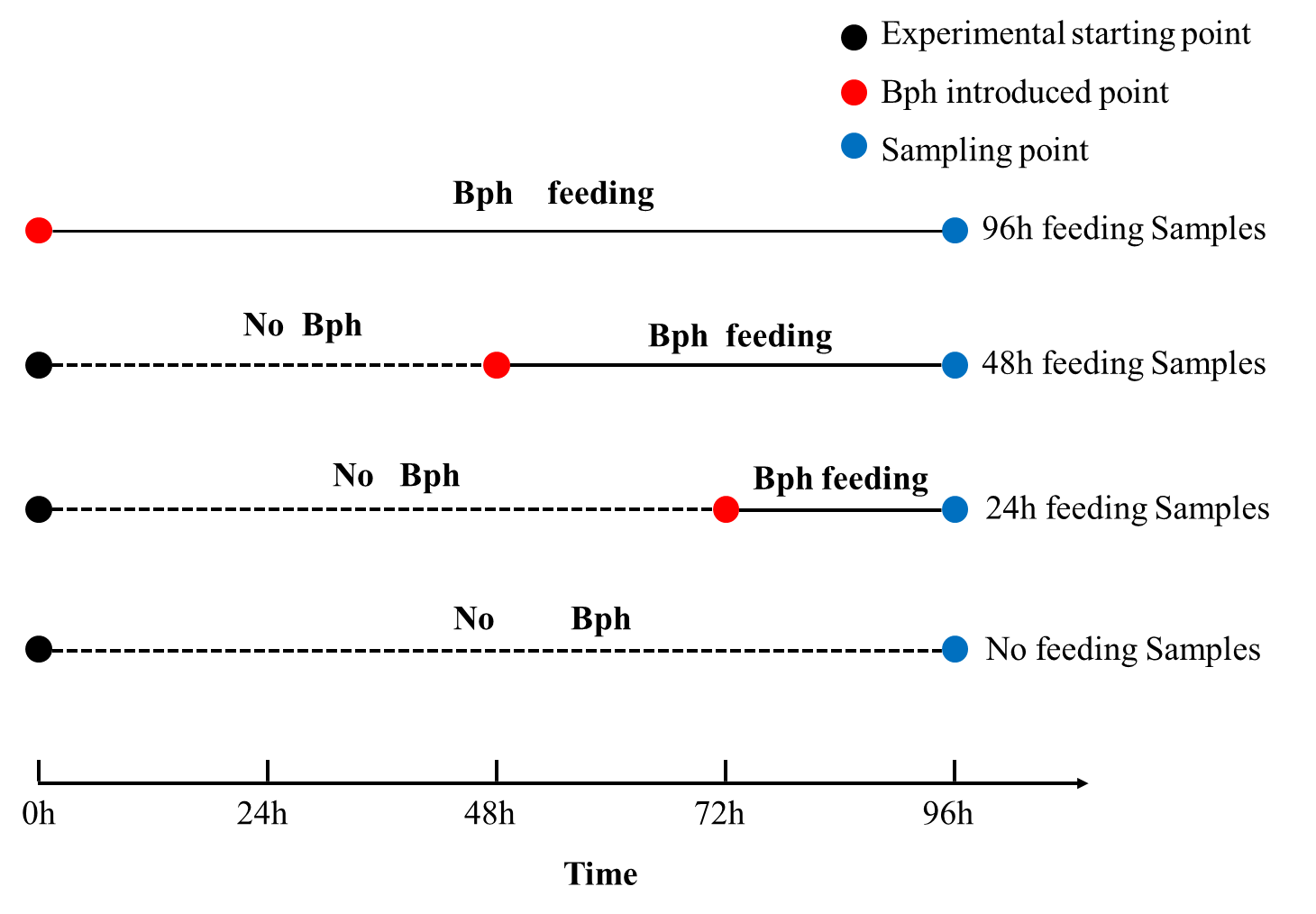
Fig. S1 The BPH feeding treatment and sampling for rice samples. One-day-old brachypterous females starved for at least 3 h prior to the start of the experiment were applied in this study. Approximately 100 adult BPH insects (*N. lugens*) were introduced to per pot (an average of 10 adults per seedling) at 0 h (96h BPH feeding samples), 48 h (48h BPH feeding samples) and 72 h (24h BPH feeding samples) after the beginning of the experiment. All the rice samples (rice leaf sheath）were collected uniformly at 96 h after the first introduction of BPH. Six biological replicates for each experimental treatment.


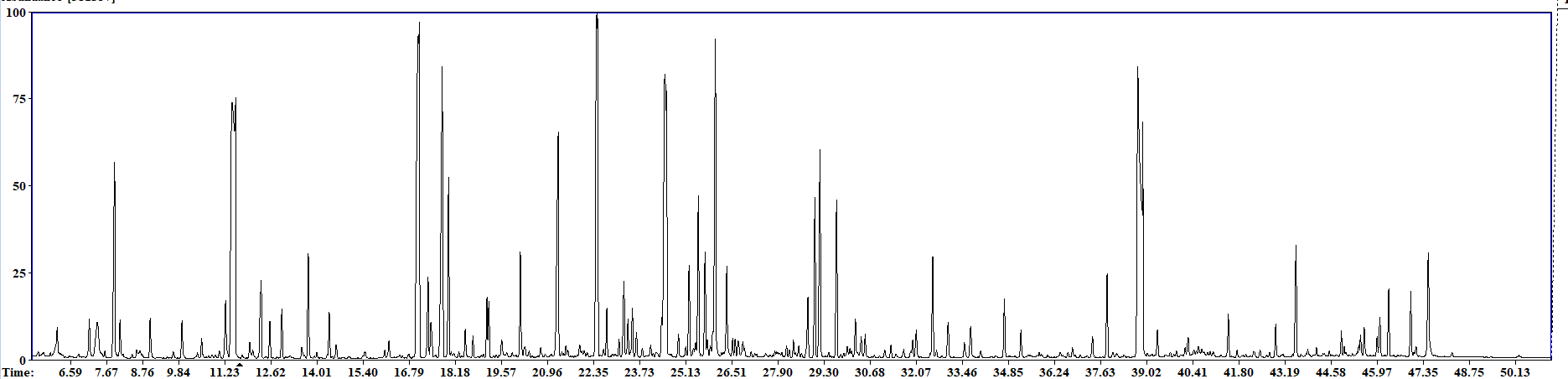


Fig. S2 GC–MS total ion chromatograms (TIC) of rice samples. TIC was acquired and exported by the Agilent MSD Chemstation (version E.02.00.493), and retention time alignment was made using the online website of XCMS (https://xcmsonline.scripps.edu/) with the default parameters.


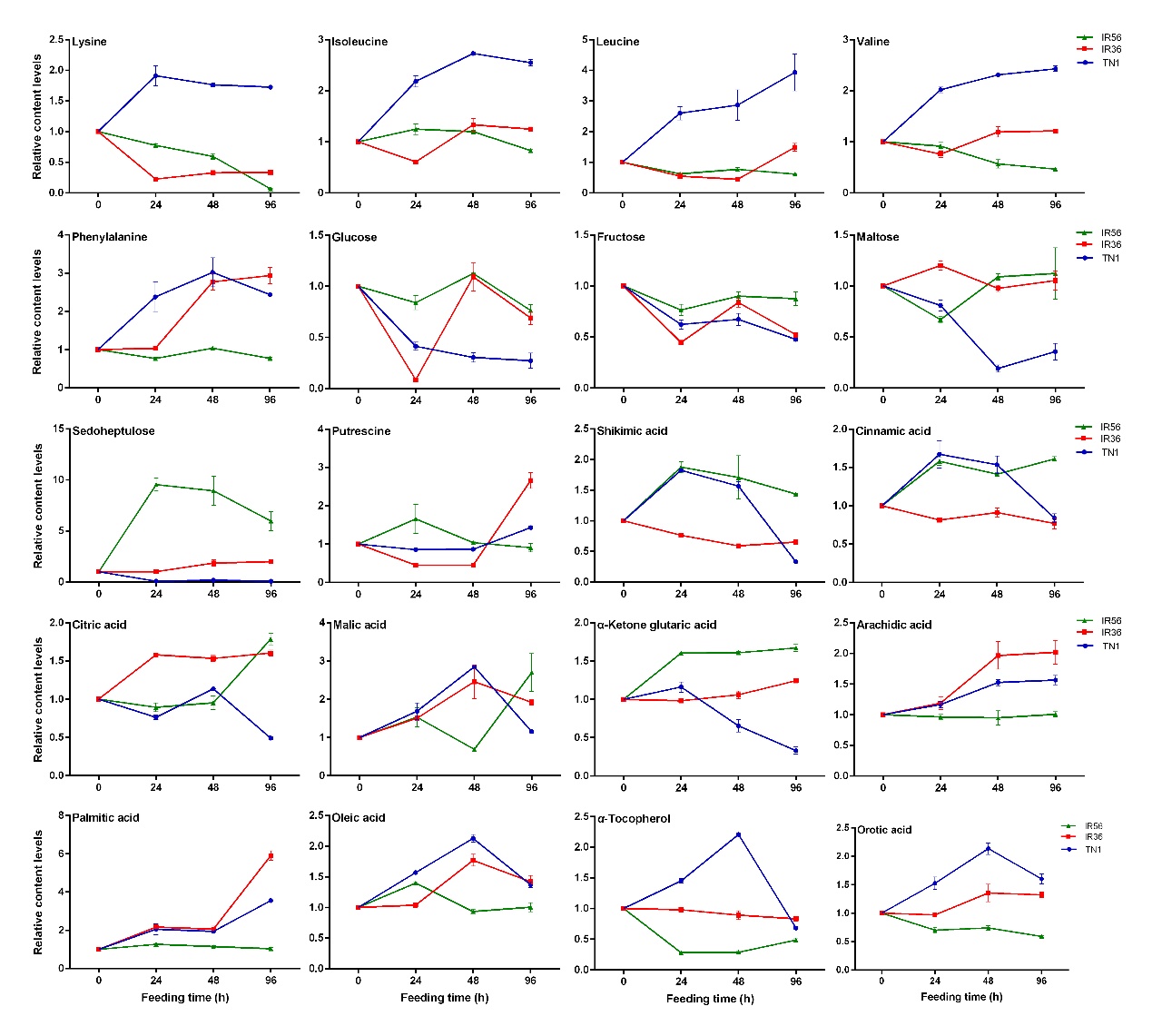


Fig. S3 Changing patterns of some metabolite levels induced by BPH feeding in three rice varieties. The relative contents of rice metabolites at 24 h, 48 h and 96 h post-BPH feeding were quantified relative to the values at 0 h (without BPH feeding) in the respective rice varieties. The green line represents the IR56 rice variety, the red line represents the IR36 rice variety, and the blue line represents the TN1 rice variety.
